# Large Left Atrial Myxoma Presenting as Functional Mitral Stenosis: A Multimodal Imaging Case Report

**DOI:** 10.7759/cureus.87401

**Published:** 2025-07-06

**Authors:** Esperance M Madera, Elda Mullaj, Anish Munagala, Gustavo Espinoza Mercado, Neeta Reddy, Induja Rajendran, Daniela Filip Kovacs, Anura Manandhar, Prakash Oli

**Affiliations:** 1 Internal Medicine, Mount Sinai Hospital, Chicago, USA; 2 Cardiology, Mount Sinai Hospital, Chicago, USA; 3 Internal Medicine, Chicago Medical School, Rosalind Franklin University of Medicine and Science, Chicago, USA

**Keywords:** atrial mass, cardiac mass resection, cardiac myxoma, echocardiogram, functional mitral stenosis

## Abstract

A 71-year-old woman with no prior cardiac history presented to the cardiology clinic following referral from her primary care physician for evaluation of progressive dyspnea, fatigue, and palpitations over a six-month period. Transthoracic echocardiography revealed a large, mobile mass attached to the interatrial septum measuring 6.4×3.9 cm, consistent with a left atrial myxoma. There was evidence of left ventricular inflow obstruction, with a mean mitral valve gradient of 15 mmHg. Further assessment determined that the mass was causing functional severe mitral stenosis, resulting in impaired left ventricular filling. A transesophageal echocardiogram confirmed the presence of the mass. The patient subsequently underwent surgical resection of the tumor via cardiopulmonary bypass. Intraoperative findings confirmed a left atrial myxoma measuring 7.1×7×4 cm. This case highlights a rare presentation of a rapidly enlarging atrial myxoma leading to functional mitral stenosis.

## Introduction

Cardiac myxomas are benign primary cardiac tumors that can originate from any chamber of the heart, though they most commonly arise from the left atrium, particularly at the fossa ovalis region of the interatrial septum [1]. While their precise prevalence is uncertain, autopsy studies estimate an occurrence of approximately 0.03% in the general population [2]. These tumors are more frequently observed in middle-aged individuals, with a higher incidence in women, although cases have been reported across all age groups [1].

Despite their benign histology, cardiac myxomas are clinically significant due to their potential to cause mechanical obstruction, valvular dysfunction, and systemic embolization, all of which can be life-threatening [3]. Large, mobile left atrial myxomas may prolapse through the mitral valve during diastole, leading to functional mitral stenosis, reduced left ventricular filling, elevated left atrial pressures, and symptoms such as dyspnea, pulmonary edema, or syncope [4]. In addition, their friable structure predisposes to embolization, which may present as stroke, renal infarction, or peripheral ischemia [5]. Some patients may also experience constitutional symptoms (e.g., fever, fatigue, weight loss), attributed to elevated interleukin-6 (IL-6) levels produced by tumor cells [6].

Clinical presentation varies widely. While some patients remain asymptomatic, others present with chest pain, dyspnea, arrhythmias, or embolic events, depending on tumor size, location, and mobility [7]. Symptoms may be positional, due to dynamic obstruction across the atrioventricular valves, and cardiac auscultation may reveal a "tumor plop" or murmurs [3].

Accurate diagnosis requires multimodal imaging, given a differential that includes thrombi, vegetations, and malignancies. Transthoracic echocardiography (TTE) is the initial imaging modality due to its accessibility and bedside applicability. However, transesophageal echocardiography (TEE) offers superior spatial resolution and sensitivity, particularly for masses near the atrial septum [8]. Cardiac magnetic resonance imaging (MRI) allows for better tissue characterization and helps distinguish myxomas from thrombi or malignant tumors, while computed tomography (CT) is useful for surgical planning and coronary artery evaluation [9]. Prompt surgical resection is the definitive treatment, with an excellent prognosis when complete resection is achieved [3].

## Case presentation

A 71-year-old woman with a past medical history of prediabetes and hyperlipidemia presented to the outpatient cardiology clinic with a six-month history of progressive fatigue, exertional dyspnea, and palpitations. She denied chest pain, orthopnea, paroxysmal nocturnal dyspnea, or lower extremity edema.

On presentation, vital signs were notable for a heart rate of 86 beats per minute, a respiratory rate of 18 breaths per minute, a blood pressure of 92/56 mmHg, and an oxygen saturation of 97% on room air. Cardiovascular examination revealed a regular rate and rhythm with normal S1 and S2 heart sounds and no S3, S4, or audible murmurs. Lung auscultation was clear bilaterally. The remainder of the physical examination was unremarkable.

**Figure 1:**
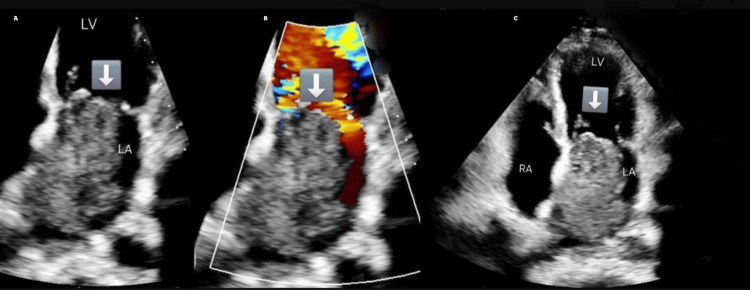
Multimodal imaging of transthoracic echocardiographic findings in left atrial myxoma (A) Two-chamber view showing a large left atrial mass with evidence of left ventricular inflow obstruction with a mean pressure gradient of 15 mmHg. (B) Apical four-chamber view showing a large left atrial mass attached to the interatrial septum. (C) Two-chamber view showing a large mass in the cavity of the left atrium

An electrocardiogram (ECG) showed normal sinus rhythm, and a 24-hour Holter monitor was obtained to assess for intermittent arrhythmia. A TTE demonstrated a large, mobile mass in the left atrium, attached to the interatrial septum, measuring 6.4×3.9 cm, with a mean mitral valve pressure gradient of 15 mmHg, consistent with functional mitral stenosis (Figure 1A-1C).

**Figure 2:**
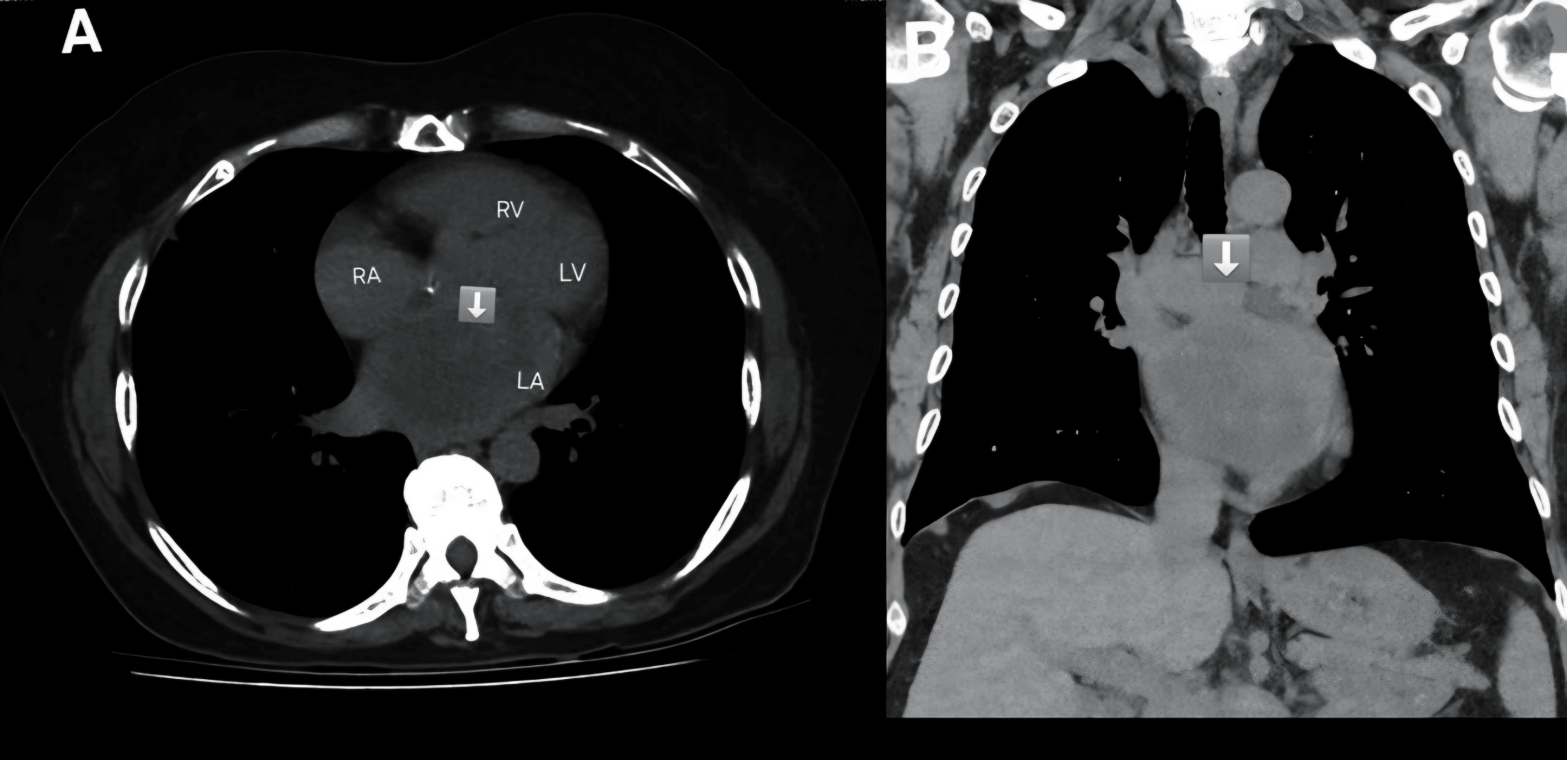
Bimodal CT assessment of left atrial myxoma (A) Axial CT view showing a solid tissue mass, concurrent with a hypodense lesion in the left atrium. (B) Coronal CT view showing a large hypodense lesion in the left atrium CT: computed tomography

A non-contrast chest CT scan confirmed the presence of the mass, measuring 6 cm in maximum dimension (Figure 2A-2B). Incidentally, a solid pulmonary nodule was noted in the right lower lobe, which on follow-up evaluation was deemed clinically insignificant and unrelated to the cardiac mass.

**Figure 3:**
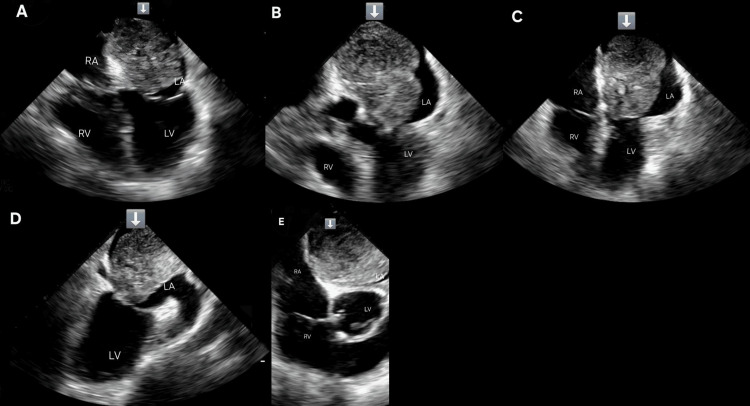
Multimodal images of transesophageal echocardiographic assessment of left atrial mass (A-C) Mid-esophageal four-chamber view showing the myxoma in the left atrium. (D) Mid-esophageal long-axis view showing the myxoma in the left atrium causing outflow obstruction. (E) Mid-esophageal long-axis view showing the large atrial myxoma associated with severe mitral flow obstruction, with a mean gradient of 15 mmHg

A TEE provided enhanced characterization of the lesion, revealing a 5.1×3.4 cm, highly mobile mass attached to the interatrial septum and prolapsing into the mitral valve, consistent with a left atrial myxoma (Figure 3A-3E). No thrombus was observed. Coronary angiography revealed no evidence of obstructive coronary artery disease.

The patient was admitted for cardiothoracic surgical evaluation. On admission, she was hemodynamically stable, and her physical exam and laboratory studies were unremarkable. Repeat ECG showed normal sinus rhythm, with left atrial enlargement and low-voltage QRS complexes, likely reflecting chronic inflow obstruction and pericardial fluid. A chest radiograph showed mild cardiomegaly without evidence of acute pulmonary pathology.

**Figure 4:**
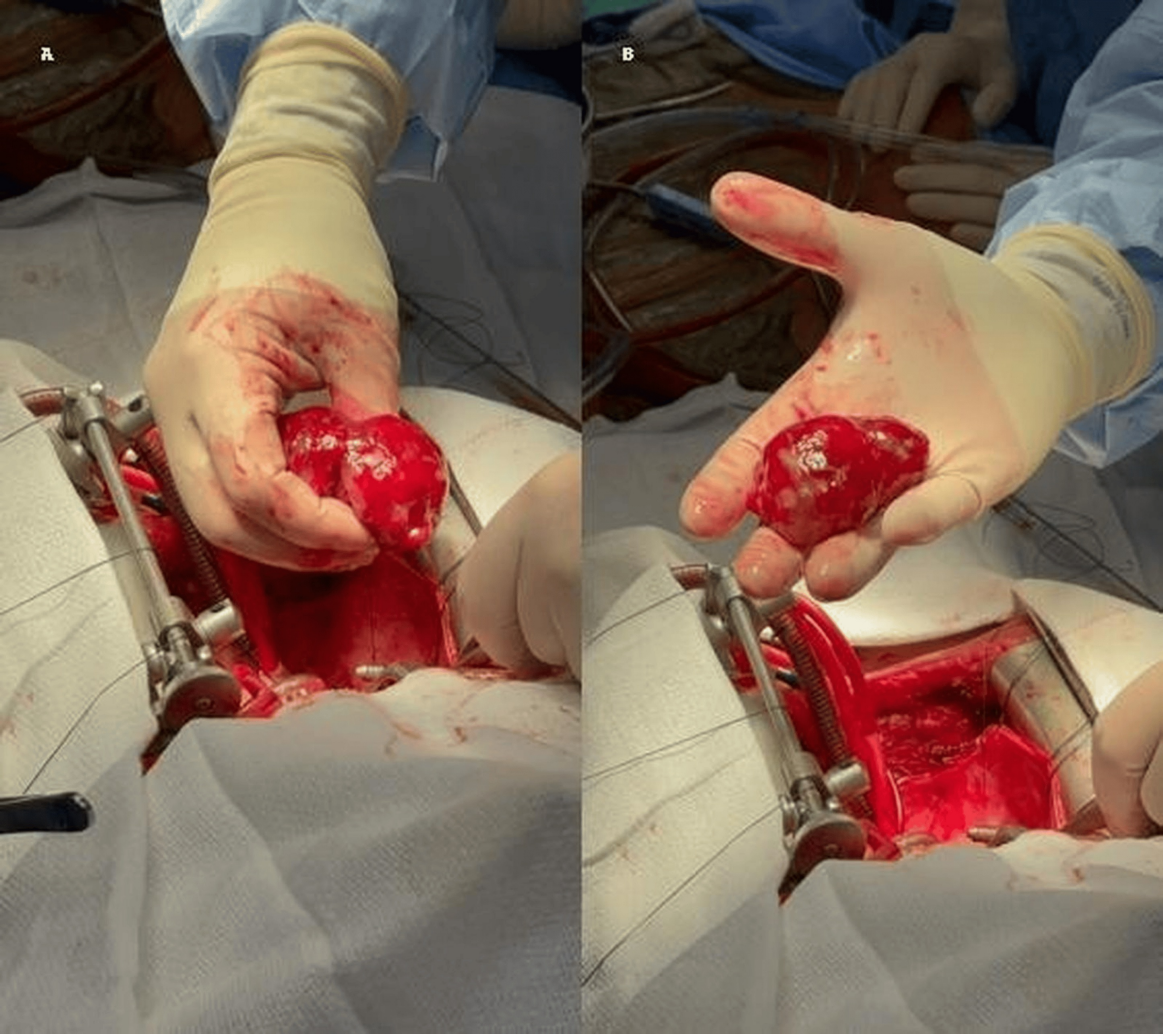
Surgical images showing median sternotomy and total cardiopulmonary bypass Resected left atrial mass measuring 8×6 cm in size along with its stalk attachment to the atrial septum

The patient subsequently underwent median sternotomy and total cardiopulmonary bypass. An 8×6 cm myxoma was resected, including its broad-based stalk attachment to the interatrial septum. Sinus rhythm was preserved intraoperatively. A bipolar ventricular pacing wire was placed, along with two mediastinal chest tubes and one right pleural chest tube (Figure 4A-4B).

Postoperative TTE confirmed complete resection of the mass and revealed a trivial pericardial effusion, without signs of tamponade. Histopathological analysis confirmed the diagnosis of myxoma, with immunohistochemical staining positive for calretinin, CD31, and CD34, markers supportive of a myxomatous origin.

The postoperative course was complicated by cardiogenic shock, which developed in the immediate postoperative period. The etiology was considered multifactorial, including transient myocardial dysfunction, volume shifts, and reduced preload following sudden relief of mitral obstruction. The patient was treated with vasopressors and transfusions, with gradual clinical improvement.

She was discharged on aspirin 81 mg daily, atorvastatin 40 mg daily, and metoprolol tartrate 12.5 mg twice daily, with instructions to follow up with cardiology and cardiothoracic surgery. Given the risk of tumor recurrence, particularly in familial cases or incomplete resections, she was enrolled in a long-term surveillance plan with repeat TTE at six months, followed by annual imaging.

Anticoagulation was not initiated, as there was no atrial fibrillation, there were no prior embolic events, and complete resection was achieved. Aspirin monotherapy was considered sufficient for secondary prevention.

## Discussion

Cardiac tumors can present with a broad spectrum of clinical manifestations, ranging from asymptomatic findings to complications such as embolization or cardiogenic shock. The diagnostic approach typically begins with a thorough clinical evaluation followed by imaging studies, including TTE and, in selected cases, TEE or cardiac MRI. Due to the broad differential, which includes thrombi, vegetations, metastatic lesions, and benign neoplasms, accurate and multimodal imaging is critical for diagnosis and surgical planning.

Primary cardiac tumors are rare, with an autopsy incidence between 0.001% and 0.03%, whereas secondary (metastatic) cardiac tumors are estimated to be 30 times more common [1]. Among primary cardiac tumors, approximately 75-85% are benign, with atrial myxomas being the most frequent in adults, comprising nearly 50% of benign cases [1,3]. Other benign tumors include rhabdomyomas, fibromas, lipomas, papillary fibroelastomas, hemangiomas, and cystic atrioventricular node tumors.

Atrial myxomas most commonly arise from the left atrium, especially near the fossa ovalis, and typically present between the ages of 40 and 60 years, with a female predominance [1]. Although most are sporadic, familial variants occur in the context of syndromes such as Carney complex. Histologically, they consist of stellate and spindle cells in a myxoid stroma, sometimes with calcifications. The tumors can be pedunculated or sessile, and friable or villous surfaces increase the risk of systemic embolization.

The clinical presentation varies based on tumor size, mobility, and location. Dyspnea is the most common symptom, often due to functional mitral stenosis when the tumor prolapses into the mitral valve. Other symptoms may include fatigue, syncope, embolic events, or constitutional complaints such as fever, weight loss, and arthralgia. On physical exam, auscultatory findings may include a systolic murmur, tumor plop, or diastolic murmur, depending on the hemodynamic effect of the mass [10].

In our patient, TTE was the initial diagnostic modality, revealing a large, mobile left atrial mass with mitral inflow obstruction and a mean pressure gradient of 15 mmHg. TEE provided superior visualization, confirming the tumor's attachment and mobility. The mass measured 5.1×3.4 cm on TEE, 6.4×3.9 cm on TTE, 6 cm on CT, and 8×6 cm intraoperatively. These discrepancies are expected due to the tumor's mobility, varying imaging planes, and differences in modality resolution. Intraoperative measurement is considered most accurate.

Mitral regurgitation (MR) was noted on TEE but could not be precisely graded due to an eccentric regurgitant jet, which limited standard assessment methods such as vena contracta width or effective regurgitant orifice area. The MR was presumed to be functional, secondary to tumor interference with mitral leaflet coaptation.

Cardiac MRI and cardiac CT were not performed due to the unavailability of the modalities at our facility and the adequacy of diagnostic clarity provided by TTE and TEE. Cardiac MRI remains a valuable adjunct in selected cases, particularly for tissue characterization, thrombus-tumor differentiation, and detection of tumor neovascularization [11]. Chest CT was performed primarily to evaluate coronary anatomy and extracardiac structures in preparation for surgery.

For patients over 40, coronary angiography is commonly performed prior to cardiac surgery to exclude obstructive coronary disease and mitigate perioperative ischemic risk. This is based on both institutional protocols and widely accepted clinical practice, although not a formal guideline mandated [12].

Inflammatory markers, including IL-6, erythrocyte sedimentation rate (ESR), and C-reactive protein (CRP), were not obtained in our case. However, elevated IL-6 has been associated with constitutional symptoms and tumor burden in myxoma patients and may have utility in monitoring recurrence [6]. The absence of such testing did not alter immediate management, but it is noted as a limitation in follow-up planning.

The patient underwent urgent surgery, prompted by functional mitral obstruction and a large tumor burden. Median sternotomy and total cardiopulmonary bypass were performed, and the tumor was completely excised, including its stalk from the interatrial septum. Intraoperative valve inspection revealed no leaflet damage, and valve repair was not required. Histopathology confirmed myxoma, with positive staining for calretinin, CD31, and CD34, consistent with mesenchymal origin. Calretinin is a marker of mesothelial cells and neural tissues, and in the heart, it is characteristically expressed in cardiac myxomas, making it a supportive marker for a myxomatous origin [6]. CD34 is a stem/progenitor cell and endothelial marker, also seen in fibroblasts and vascular neoplasms; its expression in the stromal cells of cardiac myxomas supports a mesenchymal origin [13]. CD31, an endothelial cell marker typically associated with vascular tumors such as hemangiomas or angiosarcomas, may show focal positivity in areas of vascular proliferation or entrapped endothelium within a myxoma. Together, this immunohistochemical profile is most consistent with a diagnosis of cardiac myxoma, particularly when arising in the left atrium near the fossa ovalis. These markers also help exclude malignant neoplasms such as angiosarcoma or mesothelioma and support the benign nature of the lesion [6,13].

The postoperative course was complicated by cardiogenic shock, which developed within 24 hours of surgery. This was presumed to be multifactorial, likely due to myocardial stunning, volume redistribution, and abrupt hemodynamic changes following tumor resection and relief of mitral obstruction. The patient responded to vasopressors and blood products, with gradual improvement.

Given the risk of recurrence, reported at 1-5%, especially in familial cases, surveillance with TTE was planned at six months postoperatively and then annually. The patient was discharged on aspirin monotherapy, as there was no atrial fibrillation, no thromboembolic history, and complete tumor resection.

Outcomes following surgical resection of cardiac myxomas are generally excellent. In a 16-year single-center study from China, 97% of patients required no repeat surgery, with five- and 10-year survival rates of 98% and 96%, respectively [14]. While these results are promising, generalizability may be limited due to differences in patient demographics, surgical expertise, and institutional volume.

## Conclusions

This case underscores the importance of considering cardiac myxomas in the differential diagnosis of patients presenting with unexplained dyspnea and signs of mitral stenosis, particularly when imaging reveals atrial masses. The use of multimodal imaging, including TTE and TEE, was pivotal in rapidly identifying the mass, characterizing its anatomical features, and guiding timely surgical planning. Early recognition and intervention based on comprehensive imaging contributed to a favorable clinical outcome, highlighting the critical role of imaging in both diagnostic accuracy and surgical decision-making.
